# Supplementary material for: Positive outcomes among nursing home caregivers in Spain during the COVID-19 pandemic: A qualitative interview study
Source: PLoS One. 2025 Apr 23;20(4):e0320663. doi: 10.1371/journal.pone.0320663 (PMC12017497; doi:10.1371/journal.pone.0320663)
Supplement: S1 File — (DOCX) [file pone.0320663.s001.docx]

**Supplementary material 1. Semi-structured interview**

**Work context and your role in the center**

1. What is your role in this center and your personal/professional background in working with the elderly?

***Questions focused on the content of their tasks:***

1. How many years have you been working at this center? And in other nursing homes or senior centers? Always in the same position? Or have you been involved in other tasks?

***Questions about their working conditions:***

1. How many days/hours do you work, on which shifts, and under what job stability conditions?
2. Are you satisfied with your job?
3. What do you value most about your job?

Look for possible aspects that may arise such as autonomy, feedback, support, ...

**COVID**

1. What experience have you had with COVID?

Were you working at the center during the first wave? Can you tell me a bit about what happened those weeks? How did you experience it (fear of getting infected, infecting the residents, infecting your family; tough decisions: whether or not to take residents to the hospital where they might die alone)?

1. If you were coming to work at the center, how did your closest environment react?
2. Did you feel supported and understood by your colleagues? And by your family?
3. Do you think there has been special recognition of the work done by nursing home staff (by society and your closest environment)?
4. How do you think they should have recognized it?

**NEGATIVE ASPECTS EXPERIENCED DURING THE PANDEMIC**

1. How would you say COVID has impacted your life? What problems or challenges have you faced? How did they make you feel?

Monitor if they mention: Overload, Depression, Anxiety, Need for psychotropic drugs, Relationship with the elderly, Death

Check if they are talking about the lockdown or after. If there has been any change.

**POSITIVE ASPECTS**

1. Would you say there has been any positive aspect of the situation you've experienced? / Can you see any positive aspect or consequence of the situation we have and are still experiencing?
2. Do you think the effort and dedication of recent times have been worth it?
3. Is there something that surprised you about yourself during this time? Something you wouldn't have believed you were capable of doing or facing before?
4. Do you believe that, with the experience gained, you can face the challenges you now have at hand in the center?

Monitor if they mention: Social support, Forgiveness, Gratitude, Acceptance

1. After everything you've been through, what would you say to: 1. Residents; 2. Colleagues; 3. Society
2. How would you describe yourself at that initial moment and how are you now? What has helped you?
