# Supplementary material for: Positive outcomes among nursing home caregivers in Spain during the COVID-19 pandemic: A qualitative interview study
Source: PLoS One. 2025 Apr 23;20(4):e0320663. doi: 10.1371/journal.pone.0320663 (PMC12017497; doi:10.1371/journal.pone.0320663)
Supplement: S2 Table — (DOCX) [file pone.0320663.s002.docx]

**Supplementary material 2. Coding manual**

| CATEGORY: WORK CONTEXT AND ROLE IN THE CENTER | |
| --- | --- |
| 1. What is your role in this center and your personal/professional trajectory in relation to working with the elderly? | - Profession (professional role in the center) - Career (Professional trajectory with the elderly) |
| *Questions focused on the content of your tasks:* |  |
| 1. How many years have you been working at this center? And in other residences or senior centers? Always in the same position? Or have you dedicated yourself to other tasks? | - Time (time spent working with elderly people). - Time at the center (time spent working at this center). - Tasks (any task you have performed). |
| *Questions focused on your working conditions:* |  |
| 1. How many days/hours do you work, during which shifts, and under what job stability conditions? | - Work schedule (days/hours you work, which shifts, full-time or part-time, weekends) - Stability (stable job or very changing schedules) |
| 1. Are you satisfied with your job? | - Job satisfaction (satisfaction or dissatisfaction with the job) |
| 1. What do you value most about your job? | - Cohesive team ("we were more organized," "a support system, from everyone") - Generational memory (they remind me of my grandparents, who passed away or are far away) - Colleague interaction: RECEIVE support from Social support among colleagues or from the supervisor: e.g. going beyond the professional role, performing tasks that aren't typically theirs to help out) - Relationship with families (providing peace of mind to the family, or any other kind of dynamics that arise with them) - Emotional care: Attention that the professional gives to the elderly to seek their emotional well-being or in relation to their emotional discomfort. It includes providing security and empathizing. Also social support for residents, support for residents. - Physical care (attention that the professional gives to the elderly's physical needs) - Grateful/joyful response (What the elderly person gives back (gratitude, with their joy for having been attended to, smile...)) - Communication (with the elderly, with colleagues) - Working conditions (schedules, salary, family reconciliation, job stability...) |
| Examine potential aspects that may arise such as autonomy, feedback, support, ... |  |
| CATEGORY: THEIR COVID EXPERIENCE | |
| 1. What experience have you had with COVID? | - Frustration - Emotional distress: Refers to the professional's distress. "I'm very bad", "having to give unpleasant news generates a negative burden on oneself". Includes Sadness: FEELING sadness. |
| 1. Were you working at the center during the first wave? Can you tell me a little about what happened those weeks? How did you experience it (fear of getting infected, of infecting the residents, of infecting your family; difficult decisions: whether or not to take residents to the hospital where they might die alone)? | - Fear of infecting the family (I didn't want to infect my family; fear they would get infected). - Fear of getting infected. - Need for recognition (being told you've done well). - Need for words of encouragement (needing a bit of encouragement with words). - Overload/powerlessness: Lack of time to do everything necessary. Also includes powerlessness. E.g. "Wow, I was left... eh... first of all, I had no way of finding a moment, of relocating myself and saying: 'well, this is happening...', because at the same time they were calling for another person or a relative was calling to ask how their family was and I had to tell them they were doing badly... so I didn't have time to, how can I put it, recover, right? To say: 'this is a moment and it will pass', no, no, the next moment I was in another context of another family that, even if they hadn't passed away, I had to give them the bad news. I mean, there were hours that didn't end... I didn't even have time for a peaceful snack, because sometimes you didn't even have time for a snack. You felt hungry and said: 'I haven't eaten anything, I haven't stopped working'." - Grief/death: feeling of sadness for the loss of elders who have been cared for and were fond of; the people they cared for have died. Also when they talk about death. - Seeing suffering: SEEING suffering, e.g. "people suffering, crying", "causes anxiety in the family"... - Guilt: wondering if they should have made other decisions like sending an elder to the hospital in those cases where they could have, for not being able to do things better. E.g. "Of suffering... that you're giving them news they perhaps weren't expecting, but the way I was doing it was so cold... Making a call like: 'he/she has passed away'. It seems to me it's like soldiers going to war, right?" - Faith/religion - Isolation/loneliness (loss of social contacts) |
| 1. If you were coming to work at the center, how did your immediate environment experience it? | - Concern: from the environment. (how they are, what will happen to them...) - Anger (Some family members got angry with the nursing home workers who decided to keep going to work). |
| 1. Did you feel supported and understood by your colleagues? And by your family members? | - Social support can come from friends, family, colleagues. It's about RECEIVING support. It is also tagged when it is due to absence (e.g. "So, here at work even we... the people... the looks, the looks, the colleagues... we couldn't, as I say, support each other because we all had a sad look and that, wow, I think there wasn't even a joke, there were no stories to tell"). - Emotional social support (sustain/support); - Informational social support: (information they were unaware of about the situation) - Instrumental social support: (taking care of the children) |
| 1. Do you believe there has been special recognition for the work that nursing home workers have done (from society and from your immediate environment)? | - Extra compensation (bonus for overtime) - Extra vacation days - Verbal recognition (gratitude, admiration...) |
| How do you think they should have recognized it? | - (Similar to question 10 with the aspects not mentioned) |
| CATEGORY: NEGATIVE ASPECTS EXPERIENCED DURING THE PANDEMIC | |
| 1. How would you say COVID has impacted your life? What problems or difficult aspects have you faced? How have they made you feel? | - Changes at work (changes in work routines) - Changes in personal routines (changes in daily life routines) |
| - - Watch out for: Overload, Depression, Anxiety, Need for psychotropic drugs, Relationship with the elderly, Death | - Isolation/loneliness (loss of social contacts) - Fear of contagion - Fear of infecting the family (I didn't want to infect my family, fear they would get infected) - Grief/death: refers to grieving or the death of people (elderly people I cared for have died) - Insomnia (Sleep problems) - Psychotropic drugs - Stress |
| - - Monitor if they are talking about the lockdown or after. If there has been any change. |  |
| CATEGORY: POSITIVE ASPECTS | |
| 1. Would you say there has been any positive aspect of the situation you've experienced? / Can you see any positive aspect or positive consequence of the situation we have and are still experiencing? | - Improved family relationship ("positive changes in the relationship with the family, such as spending more time together", how it helped to be with the family) - Cohesive team: when all its members share the objective as a common focus, accept it as their own, and strive to achieve the desired results for everyone above their individual interests (e.g. "around March 15-20 there was a week there that was... we didn't expect what was going to happen and then suddenly all the cases... eh... then there was already a support system, a system for everyone") - Communication (greater communication between professionals, with the elderly) - Social support: colleagues (RECEIVING support from colleagues or superiors; surpassing the professional role, performing tasks not typically theirs to help out) - Social support: RECEIVING support from Friends, family (Instrumental support (taking care of the children); Emotional support (sustain); Informational support (information they were unaware of about the situation)) - Post-traumatic growth (Setting priorities, realizing what is truly important) - Joy (working with more joy, being happier) - Faith - Personal satisfaction (satisfaction for having cared for a human being as best as possible) - Job satisfaction (satisfaction or dissatisfaction with the job) - Meaning: finding meaning in the task of caring for the elderly, having positive feelings towards care, care as an educator and rewarding, seeing the strengths of their work. E.g: "Yes, yes, everything you do for the good, whether it turns out well or everything you do for the good of the human being, whether it's a satisfactory result or... if you did it with good faith, with... you dedicated mmm... your maximum energies, I think it's worth it. Everything that is done for a human being is worth it, yes." - Humanization ("we became more human") - Grateful/joyful response (What the elderly person gives back (gratitude, with their joy for having been attended to, smile...)) - Communication (with the elderly, with colleagues) |
| 1. Do you believe the effort and dedication of recent times have been worth it? | - It's worth it. |
| 1. Is there anything that has surprised you about yourself during this time? Something you wouldn't have believed yourself capable of doing or facing before? | - PTG: Post-traumatic growth - PTG Accompanying death (the family wasn't there... and they passed away with me). - PTG Optimism (trying to see the positive side of life, greater relativization). - PTG Better problem handling (better organization of problems, better coping with them). |
| 1. Do you believe that, with the experience gained, you can face the challenges you now have at hand in the center? | - Self-efficacy (feeling that the acquired experience makes them more capable of facing future challenges) - Acceptance (accepting what comes and facing it) - Over-reactivity (less endurance) |
| - - Watch out for: Social support, Forgiveness, Gratitude, Acceptance. |  |
| 1. After everything you've been through, what would you say to: 1. Residents; 2. Colleagues; 3. Society | - Gratitude - Forgiveness (for what they might have said due to misinformation) |
| 1. How would you describe yourself at that initial moment and how are you now? What has helped you? | - Resilience (I have a greater capacity to endure. Coming out stronger from the situation e.g. "I believe we come out stronger") |
