# Supplementary material for: Positive outcomes among nursing home caregivers in Spain during the COVID-19 pandemic: A qualitative interview study
Source: PLoS One. 2025 Apr 23;20(4):e0320663. doi: 10.1371/journal.pone.0320663 (PMC12017497; doi:10.1371/journal.pone.0320663)
Supplement: Attachment — Submitted filename: Response to Reviewers FV.docx [file pone.0320663.s005.docx]

Dear Editor,

Thank you for the constructive comments about the article: "Positive outcomes among nursing home caregivers in Spain during the COVID-19 pandemic: A qualitative interview study".

We did our best and we hope our responses to your suggestions as well as the 2 reviewers' comments will be satisfactory.

Sincerely,

Dr. Ángela Ordóñez-Carabaño

**EDITOR'S COMMENTS:**

1. **The transparency and methodological robustness will be increased by paying close attention to the suggested revisions such as providing greater clarity regarding how participants were sampled and recruited**

Thank you for your guidance on improving the transparency and methodological robustness of our study. We have revised the manuscript to include a more detailed description of the sampling and recruitment processes, as suggested. In particular, we have clarified the purposive sampling approach used to select participants across different roles in both public and private nursing homes. We also provided additional details on the recruitment strategy, which included initial contact through nursing home managers and direct follow-up with interested staff to confirm their voluntary participation. These revisions can be found in the methods section on lines 169-173: “The recruitment process began with the directors of nursing homes, who informed caregiving staff about the study and provided information about voluntary participation. Caregivers who expressed interest shared their contact details, and the research team reached out by telephone to confirm participation, discuss preferred interview format, and schedule a convenient time.”

2. **I would also value a discussion of the themes that you did not include - those that were reported by less than 40% of the sample. 40% of the population is still a significant number. I look forward to reading your revised paper.**

Thank you for this insightful suggestion. In our thematic analysis, we prioritised themes that met a higher threshold of recurrence and relevance to ensure a focused and coherent presentation of the core experiences reported by carers. Themes mentioned by less than 40% of participants were considered, but did not meet the pre-established criteria of saturation or central relevance to the study objectives. Although these themes were meaningful, they did not provide additional insights that would deepen our understanding of the caregiving experience within the scope of this study.

To clarify this approach, we have added a discussion to the manuscript outlining our thematic inclusion criteria and the rationale for focusing on themes reported by a larger proportion of the sample. These changes can be found on lines 337-343: "In the following sections, we'll focus on issues that were emphasized by more than half of the participants. Following qualitative research principles, we included themes reported by at least 40% of participants, as these represent shared experiences that add depth and coherence to the analysis. Topics mentioned by fewer participants were considered non-saturating and thus were not included in the main findings, aligning with the "winnowing' approach", which emphasizes distilling the data to central, representative themes that illuminate key aspects of participants' experiences (Wolcott, 1994, cited in ^17^)."

**REVIEWER COMMENTS:**

**REVIEWER: 1**

1. **The focus of the study is appropriate, as it highlights that, in addition to intense challenges, caregivers may find positive meanings in their experiences, as indicated by previous studies. However, there is a point that could be refined. The phrase "positive aspects may mitigate negative outcomes" can be interpreted in a superficial and potentially risky manner. While the literature suggests that positive experiences may attenuate negative impacts, it would be advisable to add a caution about the importance of not romanticising caregiving, and to acknowledge that negative impacts are not necessarily eliminated by these positive aspects. Therefore, I suggest adjusting the tone of some statements that suggest positive aspects can mitigate the negative ones, recognising that both coexist without necessarily eliminating the negative impact of caregiving, particularly in crisis situations.**

We sincerely thank the reviewer for their insightful and constructive feedback. We appreciate the important reminder to address the nuanced balance between positive and negative aspects of caregiving, especially during crisis situations.

Initially, our manuscript included a comprehensive review of caregiving's negative and positive aspects. However, due to space limitations and the aim of focusing on the more novel aspects of our findings, we prioritized the discussion of positive outcomes. We recognize that this decision may have inadvertently conveyed an overly optimistic perspective, balanced initially by a more detailed discussion of caregiving's challenges and negative impacts.

In response to the reviewer's valuable observation, we have revised the relevant sections of the manuscript to incorporate a more cautious tone and to explicitly acknowledge the coexistence of positive and negative experiences without assuming that the positive aspects necessarily eliminate or fully mitigate the negative impacts. These adjustments aim to provide a more balanced perspective and avoid any unintended romanticization of caregiving.

The first revised text can be found on p. 3, lines 85-89: "It is crucial to acknowledge that these do not necessarily eliminate the challenges or the emotional burden associated with caregiving. Recognizing this coexistence helps to avoid romanticizing caregiving and highlights the complexity of the caregiving experience, particularly in crisis situations" and lines 95-98: "This study focuses on the positive outcomes to address a less-explored aspect of the caregiving experience; however, it is important to note that this approach does not imply ignoring the equally significant challenges and negative impacts faced by caregivers."

The other modifications made to the text will be presented as responses to the additional comments related to this aspect that the reviewer has provided.

2. **The methodology was well-developed, detailed, and coherent. I only have a few remarks: Regarding the statement, "The questions were validated by a professional caregiver to ensure clarity and relevance," how was this validation conducted? Was a cultural adaptation interview performed with this caregiver, or did they simply read the questions and provide feedback? How does this strategy validate the questionnaire? What is the theoretical justification for it?**

**The validation of qualitative interview guides is typically conducted through a "pilot study," where a cultural adaptation (the technically correct term for a "pilot study" in qualitative research) is carried out to assess the researcher's approach and to test whether the guide effectively supports the data collection process for the study's objectives. The authors should explain this.**

Thank you for the positive feedback and for raising this important point. The validation process for the interview questions followed two key steps. First, a geriatric physician with extensive experience in nursing home settings reviewed the questionnaire to ensure the clarity and appropriateness of each question within the specific cultural and professional context of nursing homes. The physician assessed the questions' phrasing and relevance, particularly in relation to the terminology commonly understood by staff in nursing homes.

In addition, the first interview was conducted as a pilot, allowing us to confirm that all questions were clearly understood by participants and to make minor adjustments if needed. This piloting process is supported by qualitative methodology as a means of validating interview guides; it enables researchers to identify any potential ambiguities in the questions and to adapt them accordingly to enhance data quality. This approach aligns with methodological recommendations from experts in qualitative research, who highlight that piloting improves the reliability of interview protocols by refining questions to be clear, relevant, and contextually appropriate (Maxwell, 2013; Creswell & Poth, 2018). We believe that these steps helped ensure that the interview guide was both comprehensive and appropriately adapted to the study's cultural context and objectives.

The changes are marked in the text on lines 153-159:

"The questions were initially reviewed by a geriatric physician with experience in nursing home settings to ensure clarity and relevance within this professional context. Additionally, we conducted a cultural adaptation through a pilot interview, which allowed us to confirm that the questions were clearly understood, culturally appropriate, and effectively framed for this specific setting ^16,17^. This step enabled us to make any necessary adjustments to improve the guide's clarity and ensure alignment with the study's objectives."

3. **What recruitment strategy was used for these professionals? What was the process for sending the consent forms and obtaining authorisations? Was it done virtually? Which platforms or software were used?.**

Thank you for your question about the recruitment and consent process. Our recruitment strategy was to make initial contact with care home managers, who then passed on information about the study to professional carers. Interested professionals voluntarily provided us with their contact details so that we could contact them directly by telephone to confirm their interest, discuss their preferred interview format and arrange a convenient date and time.

The consent process was also conducted remotely. Participants were informed in advance that the interviews would be recorded, and verbal consent was recorded at the beginning of each session to document informed consent. Video conference interviews were conducted and recorded using Microsoft Teams, while telephone interviews were recorded using a tablet device. No participants opted for face-to-face interviews, so all sessions were conducted virtually. Confidentiality of all recordings was ensured through secure storage protocols in accordance with ethical standards.

See lines 169-173, 182-186:

"The recruitment process began with the directors of nursing homes, who informed caregiving staff about the study and provided information about voluntary participation. Caregivers who expressed interest shared their contact details, and the research team reached out by telephone to confirm participation, discuss preferred interview format, and schedule a convenient time.

From December 1^st^, 2021 to July 5^th^, 2022, interviews were conducted either online or by telephone, depending on participant preference. Participants confirmed their agreement to participate and for the interview to be recorded immediately prior to the start of each session. They were provided with detailed information about the study's aims, procedures, and their rights, including the voluntary nature of their participation and their right to withdraw at any time without any negative consequences. Interviews lasted between 16 and 53.14 minutes, with an average duration of 36.8 minutes. For online interviews, conducted via Microsoft Teams, the platform's recording function was used; for telephone interviews, a tablet device was used to record the audio. They were transcribed by the research team without participant review. To protect confidentiality, all recordings were securely stored and transcriptions were anonymized, with identifying information removed during transcription."

4. **If possible, I suggest including a diagram or images to illustrate the coding categorisation process. This would reduce the textual explanation and enhance readers' understanding of the process.**

We have included a coding categorization Diagram (see lines 216-221).

5. **On saturation: Saturation, in itself, is a construct that brings the formally described criteria in its concept. According to Glaser and Strauss, the authors of this concept applied to qualitative research, the criterion of saturation is automatically applied when researchers seek to "saturate" the understanding of the phenomenon being studied, to the point where the research objective is answered. Therefore, it is not a limitation of the study to not have a specific criterion; saturation is, in fact, the sample closure criterion.**

Thank you for this insightful comment on saturation. Saturation was used here as a criterion for stopping sampling, in line with qualitative methodological standards that prioritise depth of understanding over quantitative thresholds. The study was therefore designed to reach this point of saturation, with the aim of exploring in depth the experiences of carers in care homes.

See lines 270-273: "In line with the qualitative methodology outlined by Glaser and Strauss ^20^, saturation functioned as our criterion for sample closure, ensuring a thorough exploration of the phenomenon under study".

6. **The section on the Study Field could be added to the methodology. This could facilitate understanding of the results in the context within which the participants were situated, particularly as there are often important differences in healthcare work between public and private settings. This also presents an opportunity for the authors to justify: why include both public and private contexts? In terms of representativeness, it may not make sense to compare both in a qualitative study?**

Thank you for this valuable suggestion. In response, we have added a brief explanation to the methodology section to clarify the selection of both a public and a private nursing home. Our decision to include these two contexts was intended to capture a wide range of care experiences rather than to conduct a comparative analysis. Including both settings allowed us to avoid potential biases related to facility management or ownership, which may influence operational practices and care dynamics. By selecting both public and private nursing homes, we aimed to increase the diversity of perspectives without implying representativeness or direct comparison between the two. This approach is consistent with our aim to explore the experiences of nursing home staff during the COVID-19 pandemic.

See lines 132-137: "In order to capture a wide range of care experiences, we selected both a public and a private nursing home. This approach aimed to avoid potential biases related to the management or ownership of the facilities, as these factors may influence operational practices and care dynamics. However, the intention was not to carry out a comparative analysis between public and private settings, but rather to enrich the diversity of perspectives in the study. ".

7. **What is the rationale behind considering hairdressers and cleaners as caregivers? While the nature of both professions may indeed transform into caregiving, in its broader sense, the study's justification refers to healthcare work as the subject. Including categories outside of the healthcare worker context might be questioned. How do the authors justify their inclusion in this context?**

Thank you for that thoughtful observation. In residential care facilities, particularly during the COVID-19 pandemic, a wide range of staff - including hairdressers and cleaners - played a crucial role in supporting the well-being of residents. During this period, residential care facilities in Spain experienced significant shortages of both resources and staff, requiring all available staff to take on expanded roles to support resident care. Although roles such as hairdressers and cleaners are not typically classified as healthcare, in the context of the pandemic they had to provide essential support beyond their traditional duties. As reflected in the interviews conducted, all professionals within the facilities demonstrated a deep commitment to the well-being of the residents, often performing tasks that went beyond the strict definition of their job roles. This collective effort ensured continuity of care and met the increased demands for resident support in challenging circumstances. Including these roles in our study allowed us to capture this broader, collaborative approach to care that became essential in the context of the pandemic. See lines 139-146:

“A wide range of care roles were also included to gain a holistic understanding of the care experience in care homes. By interviewing people from different professional backgrounds - including medical staff, care assistants, therapists, cleaners and administrators - we aimed to reflect the interconnected and collaborative nature of care in these settings. This approach allowed us to gain insights into the unique contributions and challenges of each role, enriching our understanding of the shared and distinct experiences of the entire care team during the COVID-19 pandemic”

8. **I understand optimism as a rational process of perceiving the whole, beyond the micro-space of anguish. Recognising that the distressing moment naturally exists but should not be the sole factor influencing one's energy to act. Denying the existence of anguish is not positivity. Some authors might refer to this as toxic positivity. I suggest the authors better substantiate their analyses and interpretations based on more coherent and rigorous positivity frameworks, to avoid pitfalls such as this.**

We sincerely appreciate the reviewer's thoughtful feedback and the suggestion to strengthen our interpretation of optimism within the framework of caregiving experiences. To address this, we have revised the relevant section to provide a more nuanced discussion of optimism. These adjustments aim to avoid any unintended implications of superficial or overly simplistic positivity. We have restructured the order of certain contents (pages 10-11, lines 361-387) and added new content and references (page 11, lines 384-389): "Importantly, this optimism was not characterized as a denial of distressing moments but rather as a capacity to integrate such challenges into a broader perspective, allowing for constructive action despite adversity. This aligns with frameworks suggesting that optimism involves a balanced cognitive process that acknowledges distress while focusing on solutions and opportunities ^26^ ^27^, a process that has proven particularly relevant during the COVID-19 crisis^28^."

9. **The theme of satisfaction, gratitude, and improvement in relationships is scattered across all categories. I do not see the need for a specific category solely addressing these elements, as they do not seem to have their own identity to justify being separate categories.**

Thank you for your thoughtful feedback on the categorisation of satisfaction, gratitude and relationship improvement. Your observations are greatly appreciated and have led us to reflect deeply on this aspect of our work. However, we have decided to retain these issues as a separate category, as we believe they have distinct theoretical and practical importance in the context of our study.

Recent research highlights the profound impact of gratitude and satisfaction on psychological and relational well-being. For example, studies have shown that expressing gratitude in relationships promotes relational self-efficacy and life satisfaction, even in challenging times such as the COVID-19 pandemic (Buenconsejo et al., 2023). In addition, gratitude serves as a moderating factor in improving relationship quality and life satisfaction, particularly for older adults navigating affection and conflict in close social relationships (Richards et al., 2023). Meta-analytic evidence also highlights the consistent association between dispositional gratitude and well-being across domains, further confirming the need for specific focus on these issues (Portocarrero et al., 2020).

Given this robust body of evidence, we believe that maintaining this category provides critical insights that could be diluted if these themes were integrated elsewhere. We are confident that this decision will enhance the conceptual clarity and practical applicability of our findings.

References: Buenconsejo, J., Fincham, F., & Datu, J. (2023). The perks of being grateful to partners: Expressing gratitude in relationships predicts relational self-efficacy and life satisfaction during the COVID-19 pandemic.. *Applied psychology. Health and well-being*. <https://doi.org/10.1111/aphw.12447>.

Richards, N., Zhou, Z., & Fingerman, K. (2023). Gratitude, relationship qualities, and life satisfaction in late life. *Innovation in Aging*, 7, 673 - 673. <https://doi.org/10.1093/geroni/igad104.2189>.

Portocarrero, F., Gonzalez, K., & Ekema-Agbaw, M. (2020). A meta-analytic review of the relationship between dispositional gratitude and well-being. *Personality and Individual Differences*, 164, 110101. <https://doi.org/10.1016/j.paid.2020.110101>.

10. **However, some sections, such as the description of "satisfaction derived from participants' roles," could benefit from a clearer explanation that these positive feelings do not eliminate the emotional burden of caregiving during the pandemic. This would ensure that positivity is not interpreted as a way to downplay suffering. For this reason, I suggest delving more deeply into the distinction between healthy resilience and toxic positivity. While the cited literature extensively discusses resilience and post-traumatic growth, it would be valuable to acknowledge that, in some cases, these experiences may coexist with unresolved psychological distress, something that should be addressed to avoid superficial interpretations.**

We are grateful to the reviewer for highlighting the need to provide a more nuanced discussion regarding the positive feelings reported by participants and their coexistence with the emotional burden of caregiving. We fully agree that it is essential to distinguish between healthy resilience and potential misinterpretations. In response, we have revised the relevant section to incorporate this distinction and included additional context drawn from the literature on resilience and post-traumatic growth. The revised text can be found in p. 14, lines 549-556: "While participants expressed satisfaction with their actions and contributions, it is important to acknowledge that this sense of fulfillment coexisted with significant emotional and physical demands. Caregiving during the pandemic presented immense challenges, and feelings of pride and accomplishment did not negate the ongoing stress and burdens faced by caregivers. For instance, one participant voiced a strong sense of pride in their accomplishments and an unequivocal willingness to take the same actions again if faced with a similar situation, even while acknowledging the effort required:"

Lines 562-564: "These findings underscore the dual nature of caregivers' experiences, where professional satisfaction and pride coexist with the emotional and physical tolls of caregiving, reflecting the complex and multifaceted reality of their roles**."**

11. **Overall, incorporating a deeper analysis of how caregivers balanced resilience with emotional burden would ensure that the discussion remains critical and grounded in the reality of lived experiences.**

We thank the reviewer for their valuable feedback. We have revised the discussion to address this point in greater depth. We also integrate additional references to support this nuanced perspective. The updated discussion can be found in

p. 16, lines 652-659: "According to Tedeschi and Calhoun^26^, growth cannot occur without an initial psychological disruption that challenges pre-existing beliefs and creates the need to rebuild a sense of coherence. This process does not eliminate pain but enables individuals to find meaning and purpose even while experiencing emotions such as sadness, loss, or frustration. In this sense, post-traumatic growth does not replace or negate the negative effects of trauma; rather, both elements coexist in a dynamic balance. Individuals can experience resilience and growth while simultaneously facing symptoms such as anxiety or stress."

P. 17, lines 682-686: "These positive outcomes did not emerge in isolation from significant emotional burdens; the duality of caregiving experiences—where positive adaptations coexist with profound emotional distress—reflects the complex and multifaceted nature of caregiving during crises."

p. 17, lines 710-712: "However, it is crucial to acknowledge that resilience does not negate the emotional burdens of caregiving; instead, it reflects the ability to navigate these burdens while maintaining a sense of purpose and functionality."

p. 18, lines 716-719: "This finding underscores the complexity of resilience, emphasizing that it is not synonymous with the absence of hardship but rather a dynamic process that integrates emotional challenges with the capacity to recover and grow."

p. 18, lines 736-747: "It is important to emphasize the distinction between the focus on the positive aspects identified in our study and toxic positivity or the trivialization of suffering. For post-traumatic growth to occur following adverse events, new trauma-related information can only be processed in two ways^27^: it is either *assimilated* into existing world models (which would imply a lack of elaboration necessary for growth) or *accommodated* within those models. To transcend the pre-trauma baseline, accommodation rather than assimilation is required, as growth, by definition, involves the development of new worldviews and a significant effort in the elaboration of the traumatic experience. While the alleviation of distress can result from either assimilation or accommodation processes, only accommodation fosters growth. In contrast, assimilation may leave an individual more vulnerable to future retraumatization."

p. 19, lines 771-782: "At this point, it is essential to acknowledge the persistent emotional burdens that coexist with these positive dynamics, ensuring that interventions provide holistic support that addresses and validates the challenges caregivers face. The process of accommodation described earlier requires significant cognitive effort, and any intervention that impedes this process may prove detrimental^27^. Furthermore, adversity does not inherently lead to positive change for everyone, and it is crucial to avoid conveying that there is something inherently positive in every traumatic experience. The pressure to "overcome" trauma quickly can create feelings of isolation for those who do not experience immediate or visible growth; as Calhoun and Tedeschi (1998)^49^ state: "...it is important to use language that clearly situates the impetus for growth within the struggle against the event, not in the event itself" (p. 366)."

**REVIEWER: 2**

1. **Introduction: Due to the data collected from different professional groups in the nursing homes, more supplementary information about the context of Spanish nursing homes, how they were organized, and work assignments need to be included. A more complementary context will also give a greater understanding of how their collaboration across positions contributed to positive outcomes, which is the article's purpose. Furthermore, we know the restrictions and measures implemented differed in different countries. A brief description of what this was like during COVID-19 will also contribute to an increased understanding of the results.**

Thank you very much for your valuable feedback. We have expanded the introduction to provide more context on the organisation and roles within Spanish nursing homes, highlighting how collaboration between professionals contributed to positive outcomes during the COVID-19 pandemic. We have also included a brief overview of the specific restrictions implemented in Spanish nursing homes, such as internal confinement and visitor restrictions, to highlight the unique operational challenges faced (lines 36-66). We hope that these additions enhance the understanding of our findings, as you suggested:

" Among these, residents of nursing homes were one of the most affected groups in terms of infections and deaths, having more severe cases and deaths from SARSCoV-2 infection during the first wave than in any other setting in Spain. During the first wave the number of deaths with confirmed or suspected COVID-19 in nursing homes was almost 20.000 patients ^2^. Moreover, the effects of the crisis were far-reaching, impacting not only the residents but also their families and healthcare staff ^3^.

In Spain in 2020 there were over 5.000 nursing homes for the care of older adults with more than 380.000 beds, and important differences regarding management (public or private) and size between different regions and centers ^4^. In march of that year it was promoted the coordination between nursing homes and the health system for COVID-19 diagnosis and management, and it was enforced prevention measures in these centers, including internal confinements, visitor limitations, and enhanced access controls to protect the highly vulnerable resident population ^5^.

While necessary for outbreak control, these restrictions introduced operational complexities and required adaptation from all nursing home personnel ^6^. Additionally, these restrictive policies sometimes had unintended negative impacts on both residents and their formal and informal caregivers, highlighting the delicate balance required in crisis management within long-term care facilities ^7,8^

Moreover, this multi-layered, collaborative response was crucial for effective crisis management ^9^, as well as different factors from the centers and the residents that constrained the capacity of these facilities to meet the demands of the COVID-19 crisis ^7,10^. The collaboration among professionals helped mitigate some adverse effects of the pandemic. Research highlights that collaborative coordination enabled a faster, adaptive response to the health crisis, underscoring the importance of cross-disciplinary cooperation within nursing homes ^11^. Positive outcomes of this coordination were observed in improved resident care and reduced hospitalization rates ^12^"

2. **Method: An account is given of how informants were recruited and why having different occupational groups within the nursing homes has been desirable. It is pointed out that by interviewing different employees who have different work functions, we will get a more holistic understanding of what has been a positive outcome. It is argued that: This approach allowed for a richer and more inclusive analysis, reflecting the varied roles and experiences of the entire caregiving team.**

Thank you for highlighting the importance of clarifying our rationale for including different professional groups within care settings. We agree that capturing the diverse roles of all team members - from healthcare professionals to support staff - was essential to achieving a holistic understanding of positive outcomes in care during the COVID-19 pandemic. By interviewing people from different professional backgrounds, we were able to capture the different yet interrelated experiences that contributed to the overall care environment. Each role, from medical staff to cleaning staff, provided unique insights into how the team collectively fostered resilience, mutual support and a positive atmosphere for residents.

We have further explained this rationale in the methodology section to emphasise that the inclusion of a wide range of professional groups allowed for a richer and more comprehensive analysis. This approach is consistent with the aim of our study to understand the full experience of care and its positive effects. These revisions can be found on lines 132-146:

"We aimed to conduct interviews with a diverse sample of professional caregivers in nursing homes to explore their experiences during the COVID-19 pandemic. In order to capture a wide range of care experiences, we selected both a public and a private nursing home. This approach aimed to avoid potential biases related to the management or ownership of the facilities, as these factors may influence operational practices and care dynamics. However, the intention was not to carry out a comparative analysis between public and private settings, but rather to enrich the diversity of perspectives in the study.

A wide range of care roles were also included to gain a holistic understanding of the care experience in care homes. By interviewing people from different professional backgrounds - including medical staff, care assistants, therapists, cleaners and administrators - we aimed to reflect the interconnected and collaborative nature of care in these settings. This approach allowed us to gain insights into the unique contributions and challenges of each role, enriching our understanding of the shared and distinct experiences of the entire care team during the COVID-19 pandemic."

3. **Mixing different employee positions and then functions can at the same time, create ambiguities in the further reading of the analysis and results because their roles involve different types of responsibilities, tasks and physical proximity to sick residents, contact with relatives etc. (see the comment above about the introduction). It is pointed out that themes mentioned by less than 40% were seen as non-saturation and, therefore, not included. This opens up the question of whether the positive outcomes were more or less equally distributed across the employees or whether some occupational groups stood out. If possible, indicating in the text if there were differences would have increased the quality of the analysis and the results.**

In our study, we deliberately included professionals from a variety of roles to capture a comprehensive view of the caregiving experience, as this diversity reflects the collaborative nature of work in care homes (lines XXX). Rather than focusing on role-specific differences, we aimed to identify widely shared positive outcomes that cut across roles. This choice allowed us to highlight themes that were relevant to the entire workforce and to promote a clear, consistent understanding of the positive aspects of care that emerged during the COVID-19 pandemic.

To ensure analytical coherence, we set a threshold for theme inclusion, only including themes that appeared in at least 40% of the interviews. This approach is consistent with qualitative saturation principles by focusing on themes that represent shared, saturating experiences rather than isolated or role-specific instances (lines 275-278).

Our analysis revealed that themes such as enhanced team cohesion and resilience were widely experienced across all roles, suggesting that these positive outcomes were collective rather than restricted to specific occupational groups. This shared experience of resilience, cohesion, and growth underscores the interdependent nature of care in nursing homes during a crisis, where diverse roles collaborated closely to address unprecedented challenges.

Notably, our qualitative findings provide the basis for a quantitative study we are currently conducting, which aims to analyze outcomes across different professional roles in nursing homes in greater depth. This ongoing research will examine how specific occupational groups may experience positive outcomes differently, including potential role-specific variations in resilience, satisfaction, and team cohesion. Thus, while the present study focuses on shared themes across roles, future analyses will allow us to further investigate and quantify these differences.

4. **Although the study aims to illuminate positive outcomes, the interview guide has also asked for challenges and negative impacts that emerge as data in the Coding manual (supplementary material 2). These categories, the results, could have advantageously been highlighted explicitly both in the introduction as part of the body of former research on the challenges during the COVID-19 pandemic and in the discussion section.**

We prioritised highlighting the novel findings of the study to avoid making the article too long, as this would exceed the length required for publication. Although the main text focuses on the positive outcomes, the challenges and negative impacts were incorporated into the overall analysis and are available in Supplementary Material 2. This approach allows us to adhere to publication constraints, while ensuring the rigour and comprehensiveness of the study.

5. **You have mixed reference styles in the paper. The list of references is numbered in Vancouver style, but in some places in the text, in the analysis and discussion section, you have included the references by name and date without a number.**

Thank you for noting the inconsistency in reference styles. We have carefully reviewed and corrected all references to ensure uniformity with the Vancouver style throughout the text, including the analysis and discussion sections.

**Journal Requirements:**

1. **Please ensure that your manuscript meets PLOS ONE's style requirements, including those for file naming. The PLOS ONE style templates can be found at** [https://journals.plos.org/plosone/s/file?id=wjVg/PLOSOne_formatting_sample_main_body.pdf and /](https://journals.plos.org/plosone/s/file?id=wjVg/PLOSOne_formatting_sample_main_body.pdf%20and%20/) <https://journals.plos.org/plosone/s/file?id=ba62/PLOSOne_formatting_sample_title_authors_affiliations.pdf>

We have made some adjustments to the manuscript to meet the style requirements of the journal.

2. **We note that the grant information you provided in the 'Funding Information' and 'Financial Disclosure' sections do not match. When you resubmit, please ensure that you provide the correct grant numbers for the awards you received for your study in the 'Funding Information' section.**

Thank you for stating in your Funding Statement:

This work was supported by Comillas Pontifical University through the 2022 call for internal research projects. The research project is entitled "Positive Effects of the COVID Pandemic on Professional Caregivers of Elderly Individuals in Nursing Homes".

Please provide an amended statement that declares *all* the funding or sources of support (whether external or internal to your organization) received during this study, as detailed online in our guide for authors at http://journals.plos.org/plosone/s/submit-now. Please also include the statement "There was no additional external funding received for this study." in your updated Funding Statement.

Please include your amended Funding Statement within your cover letter. We will change the online submission form on your behalf.

This work was supported by Comillas Pontifical University through the 2022 call for internal research projects. The research project is entitled "Positive Effects of the COVID Pandemic on Professional Caregivers of Elderly Individuals in Nursing Homes" (PP2022_10).

3. In the online submission form you indicate that your data is not available for proprietary reasons and have provided a contact point for accessing this data. Please note that your current contact point is a co-author on this manuscript. According to our Data Policy, the contact point must not be an author on the manuscript and must be an institutional contact, ideally not an individual. Please revise your data statement to a non-author institutional point of contact, such as a data access or ethics committee, and send this to us via return email. Please also include contact information for the third party organization, and please include the full citation of where the data can be found.

We modified our data statement to include our ethics committee as the institutional point of contact.

4. **Please amend your list of authors on the manuscript to ensure that each author is linked to an affiliation. Authors' affiliations should reflect the institution where the work was done**

We made sure that each author is linked to an affiliation.

5. **Please include captions for your Supporting Information files at the end of your manuscript, and update any in-text citations to match accordingly. Please see our Supporting Information guidelines for more information: http://journals.plos.org/plosone/s/supporting-information.**

We added a "Supporting Information" section at the end of the manuscript.
